# Future C loss in mid-latitude mineral soils: climate change exceeds land use mitigation potential in France

**DOI:** 10.1038/srep35798

**Published:** 2016-11-03

**Authors:** Jeroen Meersmans, Dominique Arrouays, Anton J. J. Van Rompaey, Christian Pagé, Sarah De Baets, Timothy A. Quine

**Affiliations:** 1 Geography Department, College of Life and Environmental Sciences, University of Exeter, Exeter, UK; 2 INRA, InfoSol unit, F-45075, Orléans, France; 3 Geography and Tourism Research Group, Department Earth and Environmental Sciences, University of Leuven, Leuven, Belgium; 4 CECI, CERFACS – CNRS, Toulouse, France

## Abstract

Many studies have highlighted significant interactions between soil C reservoir dynamics and global climate and environmental change. However, in order to estimate the future soil organic carbon sequestration potential and related ecosystem services well, more spatially detailed predictions are needed. The present study made detailed predictions of future spatial evolution (at 250 m resolution) of topsoil SOC driven by climate change and land use change for France up to the year 2100 by taking interactions between climate, land use and soil type into account. We conclude that climate change will have a much bigger influence on future SOC losses in mid-latitude mineral soils than land use change dynamics. Hence, reducing CO_2_ emissions will be crucial to prevent further loss of carbon from our soils.

Over the last two decades, there has been a notable growth of interest in monitoring soil organic carbon (SOC) because of the increased awareness of its key role in delivering many ecosystem services^1^,^2^,^3^,^4^. In particular, the potential of the soil to act as an important regulator of climate change at the global scale through its active carbon exchange with the atmosphere is highly valued. Given turnover times in the range of years to decades, and a storage capacity higher than the atmosphere and biosphere together, any large scale change in SOC can significantly alter the overall atmospheric CO_2_ concentration on the short-term^5^,^6^,^7^,^8^. Furthermore, SOC is known to have an important positive effect on (i) crop yields, maintaining food productivity; (ii) soil aggregate stability and water retention potential, reducing soil erosion and flood risks; and, (iii) pesticide sorption capacity and microbial degradation of nitrates, reducing groundwater contamination^9^,^10^,^11^,^12^,^13^. As maintaining healthy levels of SOC are crucial to face future climate change and its related food- and water security problems, many studies have focused in detail on how land use, climate, soil type and agro-management alter soil carbon storage. Associated improved process-based understanding of SOC dynamics has resulted in multiple models (e.g. Roth C, Century, DNDC and ICBM), which have been applied across a wide range of scales^14^,^15^,^16^,^17^. Nevertheless, recent research^18^,^19^ as well as international treaties, such as the Kyoto Protocol^20^ and EU Soil Thematic Strategy^21^, underline the clear need for more accurate spatial and temporal explicit estimates of this pool in order to establish appropriate policy measures to combat climate change, land degradation and soil fertility decline threats. Although a significant attempt has been made to produce detailed maps of SOC at the national level, these studies mainly focused on current^22^,^23^,^24^,^25^ or past trends^26^,^27^,^28^ and the potential influence of climate change on past SOC trends remains debatable^27^,^29^. Since many scenarios clearly suggest that the contribution and amplitude of climate change will become increasingly stronger over the next decades, there exists a clear need to estimate the impact of these rapidly changing conditions on large scale SOC budgets. This will be crucial for mapping associated feedbacks on climate and environmental threats. A few studies investigated the potential SOC stock response during the 21^st^ century taking into account future climate or land use change dynamics across large geographical areas such as Europe^30^,^31^ or the entire globe^32^,^33^. In these studies, land use change has been claimed to be a critical component explaining future SOC changes. However, the lack of the inclusion of interaction factors between variables that have an important influence on SOC variability at small spatial resolutions (e.g. soil type, climate, land use and agro-management) and/or the integration of rather progressive assumptions of increased net primary production (NPP) (e.g. as a consequence of agro-technological advancement and fertilisation effects due to increased atmospheric CO_2_ levels or anthropogenic nutrient depositions), which are under debate in recent studies as being suggested to have a much more limited influence on future SOC stocks^34^,^35^,^36^,^37^,^38^,^39^,^40^, results in large sources of uncertainty on global estimates^33^,^41^. The latter emphasises the urgent need for high-resolution future SOC stock predictions at the national scale.

In order to capture the potential interacting effects of future climate and land use change on SOC it is necessary to simulate these changes at a spatial resolution that captures physiographically-determined variation in soil properties. This demands high-resolution simulation of both climate and land use change. Hence, in the present study spatially detailed predictions of future evolution of SOC stocks driven by climate and land use change for France up to the year 2100 were produced by combining (i) an existing model, predicting SOC in the top 30 cm as a function of soil type, climate, land use and management^42^, with (ii) a business-as-usual land use change scenario and (iii) eight different spatially explicit climate change scenarios. In the business-as-usual land use change scenario, land use change trends between urban, cropland, grassland, forest and vineyard/orchard, observed by comparing 1990 and 2000 CORINE databases at a resolution of 250 meters, were extrapolated in time-steps of 10 years up to 2100. For these 5 land uses, probability maps were made by running logistic regressions considering a large set of physical and socio-economic factors and calibrated for each of the 22 administrative regions in France individually. The probability land use maps were used as input maps by applying a multi-objective land allocation (MOLA) procedure. The 8 spatially detailed climate change scenarios used in this study were obtained after applying a weather-type based statistical downscaling of large scale climate models considering three “Intergovernmental Panel on Climate Change Special Report on Emissions Scenarios” (SRES) scenarios. More methodological details and/or results regarding the land use and climate input data produced in the context of this study can be found in the methods section.

## Results and Discussion

### SOC trends at the national scale

**Figure 1:**
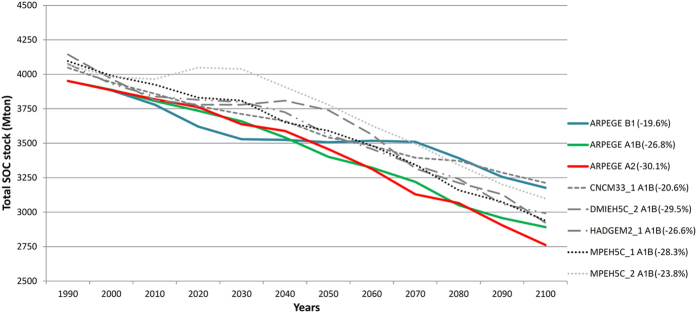
Temporal evolution of total SOC stock for entire France (Mton C) as a consequence of the combined effect of land use change and climate change considering 8 different downscaled climate models/scenarios.

Our high-resolution simulation of land use and climate change impacts on SOC stocks indicates that France will lose between 774 and 1221 Mton of SOC by 2100 (i.e. 20–30% of 1990 stock or from ca. 4,000 to 3,000 Mton C) (Fig. 1 and Table 1). More precisely, the mean SOC decrease is 1,036 ± 164 Mton C or 25.7 ± 3.9%, which corresponds with an annual decrease of circa 10 Mton C y^−1^ or approximately 12% of the total annual C emission of France, estimated in 2013 to be 82 Mton C y^−1^ 43. The most important finding of this study is that future climate change will contribute approximately 10 times more to this total SOC decrease than land use change, i.e. 966 ± 168 Mton C or 23.9 ± 4.0% for climate change versus 97.4 ± 3.7 Mton C or 2.4 ± 0.1% for land use change, respectively. We will focus in this paper on outputs of the ARPEGE model SRES scenarios B1, A1B and A2, characterized by relative SOC losses of 19.6%, 26.8% and 30.1% between 1990 and 2100 (indicated in colour in Fig. 1). Since recent research has outlined that the potential role of increased NPP forms a major source of uncertainty on large scale SOC stock trend predictions and since the associated net effect remains rather unclear^33^,^34^,^35^,^36^,^37^,^38^,^39^,^40^,^41^, this factor wasn’t considered in the present analysis. If the latter factor seems to act nevertheless as a significant negative feedback mechanism on SOC storage losses, the 21th century’s SOC losses across France may be smaller than the figures predicted in this study.

### Spatial-temporal trends in climate and land use

**Figure 2:**
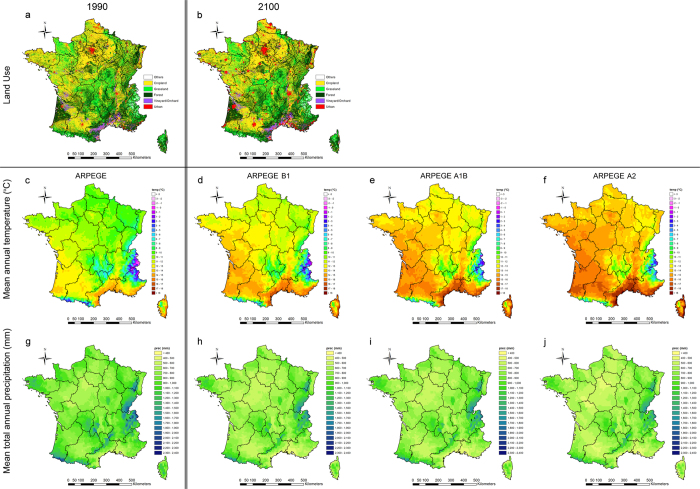
Climate and land use change maps (1990 versus 2100). Land use (**a**,**b**) and climate (i.e. temperature and precipitation according to ARPEGE model IPCC scenarios B1, A1B and A2) (**c**–**j**) input maps used for the SOC predictions of baseline year 1990 (**a**,**c**,**g**) and 2100 (**b**,**d**–**f**,**h**–**j**). The maps were generated using ArcGIS 10.1 (ESRI, Redlands, CA, USA: http://www.esri.com/software/arcgis).

The land use and ARPEGE-model derived climate variable distributions used for the SOC predictions are shown in Fig. 2 (a,b,c–j, respectively) for 1990 (baseline) and 2100. This shows that the urban sprawl will be the most important land use change process (Fig. 2a,b). The ARPEGE-model predicted temperature maps for 1990 shows that most areas have annual average temperatures below 11 °C while by 2100 most areas will have annual average temperatures above 11 °C under the SRES B1 scenario, above 12 °C under the SRES A1B scenario and above 13 °C for A2 scenario (Fig. 2c–f). Annual precipitation maps indicate a general trend of drying with most areas witnessing a reduction of 100 mm year^−1^ under SRES scenarios B1 and almost 200 mm y^−1^ under SRES scenarios A1B and A2. The predicted precipitation changes are much more pronounced in the mountainous regions than elsewhere, with absolute difference in rainfall up to 500 mm y^−1^ or more for the western part of the Pyrenees (Fig. 2g–j).

### Spatial heterogeneity of SOC change and interactions with controlling factors at the landscape scale

**Figure 3:**
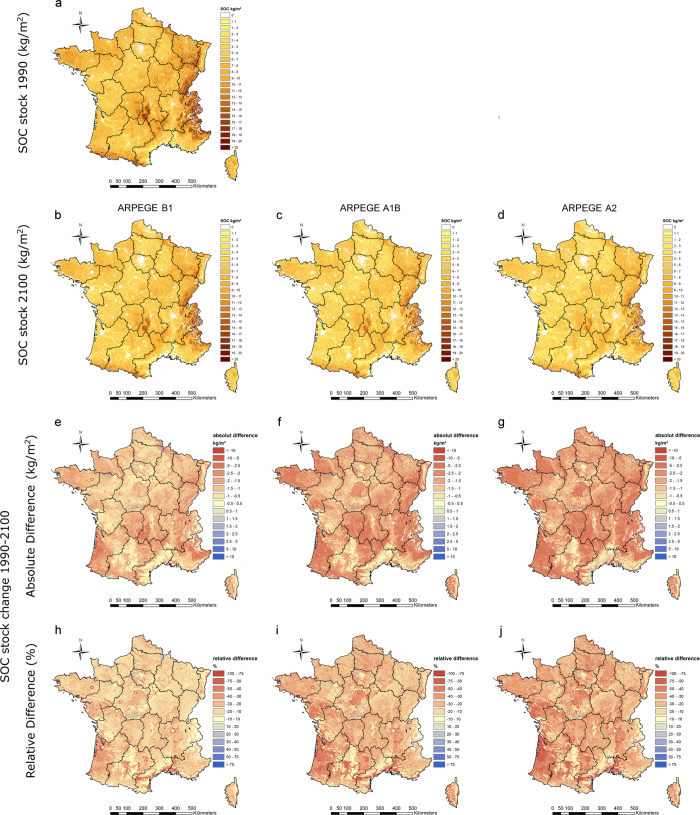
SOC change maps (1990 versus 2100). SOC stock maps (kg C m^−2^) for 1990 and 2100 as a result of ARPEGE model IPCC scenarios B1, A1B and A2 as well as land use change predictions (**a–d**), including absolute (**e–g**) and relative difference (**h–j**) maps. The maps were generated using ArcGIS 10.1 (ESRI, Redlands, CA, USA: http://www.esri.com/software/arcgis).

The baseline SOC map for 1990 shows that precipitation dominates the spatial pattern of SOC, with high stocks in wet regions such as in the south-west and most westerly part of France (Britany) and in various Mountainous regions in the east and south of France (Figs 2g and 3a). In addition this map illustrates that besides climate, land use has an important influence on the spatial distribution of SOC, with low SOC stocks in cropland areas (mostly centrally located) or vineyards (Mediterranean region) and high SOC stocks in grassland or forested areas^42^.

The future SOC maps (based on ARPEGE model) for 2100, show that SOC stocks across the entire French territory, are remarkably lower as compared to the baseline SOC map of 1990 (Fig. 3a–d). When considering SRES scenario B1, SOC losses exceeds 1 kg C m^−2^ in most areas, with a nationwide average loss of 1.43 ± 1.25 kg C m^−2^ (Fig. 3e). These losses become gradually higher under the A1B and A2 scenarios, with in most areas SOC losses exceeding 2 kg C m^−2^ and nationwide average losses of respectively, 1.97 ± 1.40 kg C m^−2^ and 2.21 ± 1.51 kg C m^−2^ (Fig. 3f,g). Nevertheless, note that there is a very large spatial heterogeneity on these nationwide SOC trends. Absolute differences are generally highest in the more wet regions (i.e. in the west and southwest of France as well in Mountainous regions in the east and south of France). Declining SOC stocks in these environments will most probably be the result of the combined effect of significantly (i) higher temperatures (Fig. 2c–f), causing elevated rates of microbial decomposition of organic matter and hence increased C-respiration^44^,^45^,^46^ and (ii) drier conditions (Fig. 2g–j), causing lower soil moisture conditions, which hampers plant growth and hence reduces C input^33^,^47^. Despite the fact that on the other hand drier conditions may results as well in a lower degree of microbial activity and thus a lower degree of decomposition, it is most probable that the decreased C input will be the dominant factor resulting in an overall reduction of SOC. This can also be observed nowadays in most Mediterranean dryland regions which are commonly characterized by very low SOC concentrations^48^.

**Figure 4:**
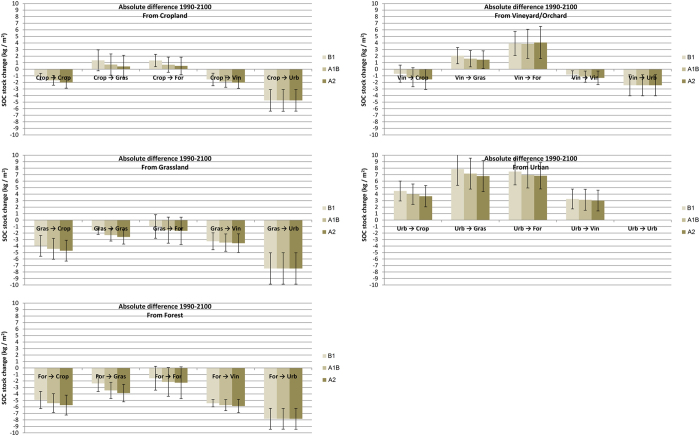
Absolute difference in SOC stock (kg C m^−2^) as a function of land use dynamics using ARPEGE model IPCC scenarios B1, A1B and A2 (standard deviations represents variability at the national scale).

**Figure 5:**
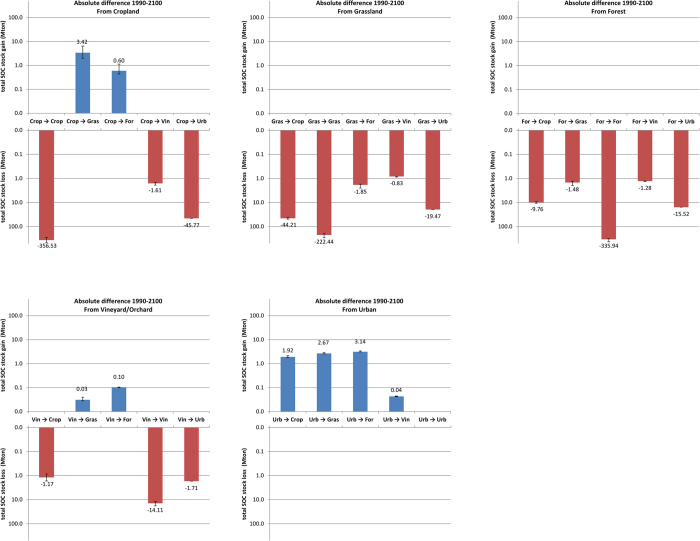
Absolute difference in total SOC stock for entire France (Mton C) as a function of land use dynamics using ARPEGE model IPCC scenarios A1B predictions (lower error bars represents ARPEGE model IPCC scenario A2 predictions, higher error bars represents IPCC scenario B1 predictions).

Furthermore, urban expansion is causing remarkably large SOC decreases near the edges of almost all existing cities (Fig. 2e–j). As urbanization causes SOC to reduce significantly over time, a decline to 0 kg C m^−2^ is assumed. Consequently, these SOC stock changes are similar for each SRES climate change scenario and as large as −4.74 ± 1.65 kg C m^−2^, −7.46 ± 2.42 kg C m^−2^, −7.83 ± 1.80 kg C m^−2^ and −2.44 ± 1.60 kg C m^−2^ for conversions from cropland, grassland, forest and vineyard into urban land use (Fig. 4). This corresponds with a total SOC stock change at the national scale under SRES scenario A1B of −45.77 Mton C, −19.47 Mton C, −15.52 Mton C and −1.71 Mton C, respectively (Fig. 5). Despite the fact that comparable SOC stock gains can be made with de-urbanisation, these are uncommon land use changes under the business-as-usual land use change scenario, and hence associate total SOC stock gains at the national scale are rather low, i.e. varying between +3.14Mton C for urban to forest conversions and +0.04 Mton C for urban to Vineyard/Orchard conversions under SRES A1B scenario (Fig. 5). Although land use change on the urban periphery is the most common and the most significant contributor to land use change related SOC change; national SOC stock change is dominated by changes in SOC stocks in areas unaffected by land use change. These SOC stock changes per unit area range from ca. −1 kg C m^−2^ for unchanged vineyard/orchard, ca. −1.5 kg C m^−2^ for unchanged cropland to ca. −2 kg C m^−2^ for unchanged grassland and forest (Fig. 4), which corresponds to relative net changes of ca. −40% for vineyards and orchards, of ca. −30% for cropland and ca. −25% for grassland and forest. Associated net contribution to the total SOC stock loss at the national scale is very large due to the vast area they occupy. Using SRES A1B scenario as an example, unchanged cropland, grassland and forest are predicted to lose 357, 222, 336 Mton C, respectively; even unchanged vineyard/orchard are predicted to lose 14 Mton C (Fig. 5). Cumulatively, these represent 86% of the predicted national total SOC stock loss due to land use and climate change, indicating that future SOC stock changes are foremost affected by climate change.

Amongst non-urban land use changes, most change combinations will results in net SOC stock losses. Largest losses are recorded for conversions from grassland and forest into cropland, estimated under SRES scenario A1B at 44 Mton C (or 4.4 ± 1.6 kg C m^−2^) and 10 Mton C (or 5.4 ± 1.4 kg C m^−2^), respectively (Figs 4 and 5). Most other land use change conversions are characterized by relatively small SOC changes with limited total stock losses (<2 Mton C). The only land use changes that result in a net gain in total SOC stock are those associated with conversions of cropland or vineyard/orchard into forest or grassland. Under the SRES A1B scenario, cropland to grassland conversion results in a net SOC stock gain of 3.4 Mton C (Fig. 5), which corresponds to a mean SOC gain of 0.7 ± 1.6 kg C m^−2^ (Fig. 4), indicating that not all areas subject to this particular land use conversion demonstrate a net gain. The SOC change maps (Fig. 3e–j) indicate that cropland to grassland conversion is most effective in storing C in the valleys in the central and northwest of France. These regions are characterized by relative low temperatures and have soils with relatively high clay contents (e.g. valley of the Seine river, Fig. 3e–j). This illustrates the important interaction between soil type and climate change in determining C sequestration potential at the national scale.

In general, sandy regions (e.g. Vosges, Landes and Sologne) are typically characterized by rather high SOC losses. The important influence of soil type on the spatial heterogeneity in SOC changes becomes also very clear in the cropland dominated region in the south of France (i.e. north of the most central part of the Pyrenees) where high SOC losses occur in soils with low clay contents and low SOC losses are predicted for soils with high clay contents. Furthermore, areas with limited SOC stock gains are stony forested soils in the Mediterranean region (Fig. 3e–j).

### Implications for land use change management impacts on SOC storage

Previous research has suggested that land use change within Europe could make a significant contribution to climate change mitigation by creating an important net SOC sink^30^,^31^. Nevertheless, the latter studies have explored the effect of widespread land use change unconstrained by local decision-making. Here we model high-resolution business-as-usual land use change based on local decisions made in the recent past and, under these conditions, we observe contrasting results. We find that the land use change conversions result in a small net soil C loss, while climate change results in a large C loss and hence will have a dominant effect on SOC stock dynamics in mineral, mid-latitude soils in the 21^st^ century. Although the conversion of urban areas into other land uses seems to be the most effective strategy to sequester C in the soil (i.e. resulting in net SOC stock gains between 3 and 8 kg C m^−2^, Fig. 4) it is not expected that there is much scope for large soil C storage here, in particular because this study shows that under a business-as-usual land use scenario urban sprawl will be the most frequent land use change during the 21st century. Moreover, as a consequence of the lack of organic matter, nutrients and stable aggregates or the potential to be polluted, it most likely will take a rather long period before soils subject to de-urbanisation will act as sustainable sinks of CO_2_. Besides de-urbanisation, only the conversions of cropland or vineyards into forest or grassland are predicted to result in a net soil C stock gain. Their potential to act as important soil C sinks is, however, strongly limited due to the small net SOC stock gains per unit area and/or the restricted land surface area that can be subject to these land use conversions.

Due to the small SOC stock gain per unit area when converting cropland to grassland or forest (i.e. only 0.5–1 kg C m^−2^), conversions of 100,000 to 200,000 km^2^ of cropland into grassland or forest would be required to offset 10% of the climate change induced loss of SOC from areas of unchanged land use. This is unrealistic as the total area of cropland in France in 1990 was only 228,000 km^2^ and maintaining cropland will be crucial to fulfil the predicted future food demand^49^, especially given the recently stagnating crop yields^40^ and potential future nutrient limitation^39^. The potential SOC stock gains related to conversions from vineyard into grassland or forest are higher (i.e. around 1.5 kg C m^−2^ and 4 kg C m^−2^, respectively (Fig. 4)), but the net total SOC stock storage potential at the national scale is small due to the restricted land surface area of vineyard/orchards. So even converting all the existing vineyards/orchards into forest, will only lead to a net sequestration of 50 Mton C, which is only 5% of the total estimated SOC stock loss. Such a change is even less probable, given the economic and cultural importance of the vineyards in France.

### Global Policy implications

The implication of this study is that only radical land use change coupled with enhanced C-sequestration in productive agricultural land uses (principally cropland) has the potential to mitigate climate change. In contrast, business-as-usual land use change driven by local land use decision-making is likely to lead to significant SOC loss due to the impact of climate change on SOC stocks. Consequently, these results indicate that despite the fact that land use management may be a valid alternative to mitigate climate change on the short-term, this seems to be less effective on the long-term. The positive feedback mechanism between soil C emissions and climate change has been shown to be the main driver of declining SOC levels, which therefore will be the primary factor endangering the functioning of soil’s vital ecosystem services such as food security, aquifer quality, soil erosion protection and flood regulation. In addition and in contrast to previous studies^30^,^31^,^32^,^33^ the present research delivers SOC stock change maps at a much finer resolution (250 m) that reflects important interactions at small spatial scales. Maps at this scale are needed by policy makers when conducting targeted management strategies for combating soil fertility decline and to protect associated ecosystem services^19^. More specifically, there is scope for counteracting the predicted changes, by using these detailed maps to focus actions on areas characterized by large future SOC stock losses, such as the sandy soils or regions prone to future drying. By promoting or enforcing environment specific land and/or agro-management techniques, such as green manuring, reduced tillage, erosion prevention, smart irrigation, agroforestry and crop rotations^50^,^51^ at farm or landscape level policy makers can attempt to counter that these areas will act as large sources of CO_2_. Nevertheless, it will be essential to consider the agroecosystem environment specific settings (i.e. soil type, climate, topography, land use & management history) in each area. Recent studies have highlighted that the effectiveness of conservation tillage practices largely depends on soil type, climate and topographical conditions as well as on crop rotation and the land use management history of the plot^52^,^53^. Moreover, despite the well understood fact that soil erosion causes a depletion of SOC at eroded sites and an enrichment at depositional sites, its net influence on soil-atmosphere CO_2_ exchange is still subject of scientific debate^54^,^55^. The potential of erosion management related measures in order to enhance soil C sequestration across larger scales on the long term is therefore unsure. Nevertheless, recent research indicates that more promising and widely applicable soil C sequestration management measures include cover crop green manuring^56^ as well as agroforestry^57^. In conclusion, our study suggests that if no action will be taken to strongly reduce global CO_2_ emission levels and in addition modify land use and soil management practices at the short-term and more drastically at the long-term, soils will act as very large sources of CO_2_ by the end of the century.

## Methods

### Land Use Change Modelling (France 1990–2100)

We conducted future land use change predictions for each of the 22 main administrative areas in France separately, covering the period 1990–2100 following a temporal grid of 10 years. Input data were derived from CORINE land cover maps of 1990 and 2000 with a resolution of 250 m^58^. These 2 land use maps were reclassified in 5 general land use classes in accordance with the requirements of the SOC model^42^ used in this study, i.e. cropland, grassland, forest, vineyard – orchard and urban. Land use change trends observed in the period 1990–2000 were extrapolated in time up to 2100 assuming a *business-as-usual scenario* (i.e. considering a constant amount of pixels changing between two given land uses for each time window of 10 years).

A logistic regression model (Equation 1)^59^ was calibrated for each of the 22 regions to produce a probability map that expresses for each generic land use class the likeliness of occurrence of a certain land use as a function of a wide set of (i) topographical, (ii) climate, (iii) soil-physical and (iv) socio-economic variables considering the 1990 land use status. More precisely.

1. Height above sea level (m), slope steepness (%), deviation of the orientation of the slope towards the North (°) (i.e. N = 0°; NW & NE = 45°; W & E = 90°; SE & SW = 135°; S = 180°) maps at a resolution of 90 metres were derived from “SRTM Digital Elevation Data”, distributed by “The CGIAR Consortium for Spatial Information (CGIAR-CSI)” and produced by NASA originally^60^.
2. Yearly averaged temperature (°C) and total precipitation (mm yr^−1^) map were obtained from a 0.125° × 0.125° climatic grid covering entire metropolitan France distributed by Meteo-France^24^.
3. Soil’s top 30 cm clay, silt and stone content (%) as well as geometric mean particle size (Dg, mm) and bulk density (g cm^−3^) maps, after combining the 1/1,000,000-scale Soil Geographical Database of France (i.e. based on the European Soil Map) and the French national soil inventory database (i.e. DoneSol 2.0, N = 17,484)^24^.
4. ARCGIS software was used to map distance to the nearest main route (i.e. classified as “Motorway” or “National route” following the French road network system) as well as population and employment potential, using statistics provided by the National Institute for Statistics and Economic Studies^61^ and spatially interpolated using an inverse distance weighted interpolator module.

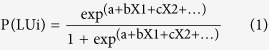


*where P(LUi)* = *Probability of land use type i; a, b, c,…* = *model parameters; X1, X2,…* = *model variables.*

When performing the logistic regression, a calibration procedure was modeled in R, using the corrected Akaike information criterion (AICc)^62^ and the requirement to have all significant parameters (p < 0.05) as model selection criteria in order to obtain the best model by applying an adapted version of the traditional stepwise regression procedure. The latter deals with the issue when the model, following a traditional stepwise regression procedure, ends-up in a local minimum (i.e. no lower AICc have been obtained when adding or removing 1 variable, but which isn’t the ultimate best model with respect to the given set of variables), by adding or removing more than 1 variable a time. This way the opportunity has been offered to go a few steps back or further at once in order to move out the local minimum, and hence, potentially find a better model (i.e. lower AICc).

The required number of pixels for each land use, as determined by the *business-as-usual* extrapolation of the 1990–2000 observed land use change trends, has been spatially allocated using the probability maps for each land use as input to run a multi-objective land allocation (MOLA) procedure in IDRISI-software in order to deal with potential conflicts of competing land uses^63^,^64^. A detailed spatial analysis of the independent variables of the logistic regression as well as a validation assessment can be found in the supplementary information file.

### Downscaling spatial explicit climate change prediction (France 1990–2100)

The climate change scenarios are based on projections of several large-scale climate models. To regionalize these scenarios, a statistical downscaling method developed by CERFACS was used. The method is calibrated using the recent climate figures, as described by the Météo-France SAFRAN meteorological analysis^65^.

The statistical downscaling method is based on weather typing, and is called dsclim. The large-scale circulation, here defined by the mean sea-level pressure and the temperature averaged over Western Europe (predictors), is hereby statistically linked to local-scale climate variables (predictants). The statistical model is established over a learning period (1981–2005), whereby the predictors are extracted from the daily NCEP reanalysis, while the predictants are taken from the SAFRAN analysis. The downscaling consists of conditional resampling, by matching the day of the large-scale climate scenario to the day of the SAFRAN analysis with the same weather type and the closest mean sea level pressure and Western Europe averaged temperature. The four standard seasons are processed separately, as different large-scale weather types characterize them.

This method satisfactorily describes the regional climate variability at a daily to inter-annual time basis, as shown for instance by the distributions of precipitation. Under the assumption that the model errors are stationary, it allows to account for the changes in climate variability in addition to mean climate change, and thus tackles the evolution of precipitation and temperature extremes.

To address the uncertainties related to the downscaled climate scenarios, we classically multiplied the latter ones to generate an ensemble of scenarios. We selected 8 of them, characterized by 5 different large-scale climate models, and downscaled by using the method described above. The ARPEGE model simulations have been performed under the European project FP6-CECILIA, and are forced by three “Intergovernmental Panel on Climate Change Special Report on Emissions Scenarios” (SRES) scenarios^66^ (i.e. B1, A1B, A2). The other climate simulations have been performed under the European FP6-ENSEMBLES considering only A1B SRES scenarios. Following climate model simulations - emission scenario combinations have been used in this study:

- ARPEGE (SRES B1)
- ARPEGE (SRES A1B)
- ARPEGE (SRES A2)
- CNCM33 member 1 (FP6-ENSEMBLES SRES A1B)
- DMIEH5C member 2 (FP6-ENSEMBLES SRES A1B)
- MPEH5C member 1 (FP6-ENSEMBLES SRES A1B)
- MPEH5C member 2 (FP6-ENSEMBLES SRES A1B)
- HADGEM2 member 1 (FP6-ENSEMBLES SRES A1B)

Finally, climatological mean annual precipitation (mm) and temperature (°C) maps for each 10 year time step (from 1990–2000) were created by calculating an average temperature and precipitation value for each grid cell from a time window of 25 years previous to the considered time step. This strategy was chosen in order to account as best as possible for the inertia of SOC upon altered climate conditions (note that the turn-over time of SOC in temperate mineral soils is typically in the order of magnitude of a few decades).

### Soil Organic Carbon model

In the present study we have combined a novel Soil Organic Carbon (SOC) model presented by Meersmans *et al*. (2012a,b)^24^,^42^ with spatial explicit land use change (see above) and downscaled climate change (see above) predictions in order to predict the spatial-temporal evolution of SOC at decadal intervals and a resolution of 250 m.

This model has been calibrated using input data from various resources covering entire France. SOC measurements from 2,158 sites, following a regular 16 × 16 km grid gathered by the French National Institute for Agriculture Research (INRA) between 2000 and 2009 and recorded in the Réseau de Mesures de la Qualité des Sols (RMQS) soil survey^67^, were used. This regular grid sampling design ensured an excellent distribution of data amongst a wide range of soil types, land use and climate combinations. The great heterogeneity of soil types in France is reflected in this database, including sandy soils in the South-West (Podsols), fertile loess soils (Luvisols) in the North as well as various shallow soils (Leptosols) developed from calcareous rocks and dystric Cambisols developed from the weathering of different sorts of parent material^68^, resulting in a large variability in silt and clay contents^24^. Climatological data for the model was obtained from the Meteo-France 0.125° × 0.125° climatic grid covering the period 1993–2004, with average yearly temperatures varying between ca. 0 °C and ca. 18 °C and total precipitation amount varying between ca. 500 mm and ca. 2500 mm^24^. Manure application and animal excrement production statistics at departmental level were used as main agro-management input data resource^24^,^42^.

The model, predicting topsoil (0.3 m) carbon concentration in fine earth as a function of land use, soil type, management and climate data (Equation 2), was created by applying a multiple linear regression analysis using a combined forward and backward stepwise regression method. The model complexity was managed using the following model selection criteria: the Akaike information criterion (AIC), the corrected Akaike information criterion (AICc) and the Bayesian information criterion (BIC) rule^62^. In the present study, the model constructed with the AICc selection criterion was used, because BIC gave considerably lower R^2^_adj_ and RPD values (ratio of performance to deviation following a K-fold cross-validation procedure) and the AIC model had 36 parameters, of which only 30 are significant (p < 0.05), whereas the AICc model had 30 parameters that are all significant. Moreover, for very low SOC concentrations (<1%) the model error of the AICc model was remarkably smaller as compared to the AIC model^24^. Hence the AICc model captures all important trends in the data without overfitting and has been selected in order to make realistic predictions in the context of the present research. Beside linear terms, first- and second order (non-linear) interactions between the input variables were taken into consideration during model construction. The effect of land use was incorporated in the model by estimating model parameters land use independent or specific for 1, 2 or 3 land uses. A detailed overview of the different variables by term of the model can be found in Meersmans *et al*. (2012a)^42^. Subsequently, modelled SOC contents are converted into SOC stock after taking soil depth, rock fragment content and fine earth’s bulk density into account^24^. Country-wide total SOC stocks were obtained by summing organic carbon stocks for all pixels and multiplying by the pixel area.


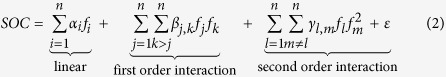


*where SOC is soil organic carbon concentration (%), n is the number of linear terms, fi is variable i, α, β and γ are model parameters and ɛ is the error term.*

Although this model approach allows incorporating all significant interactions between SOC-driving factors at the national scale, it is important to note that these interaction factors are static and therefore do not allow to account for feedbacks between the warming climate and the land surface/soil system in a dynamic context. Hence, embedding models that have the ability to incorporate these feedbacks mechanisms, such as process based models, linking the rate of change of SOC to a wide set of environmental factors (e.g. Roth C, Century)^14^,^15^,^69^, or even more sophisticated Earth System Models (e.g. JULES)^33^,^70^, in the presented methodological framework can be seen as an interesting avenue for future research.

## Additional Information

**How to cite this article**: Meersmans, J. *et al*. Future C loss in mid-latitude mineral soils: climate change exceeds land use mitigation potential in France. *Sci. Rep.* **6**, 35798; doi: 10.1038/srep35798 (2016).

**Publisher’s note**: Springer Nature remains neutral with regard to jurisdictional claims in published maps and institutional affiliations.

## Supplementary Material

Supplementary Information

## Figures and Tables

**Table 1:** Total SOC stock change for entire France (Mton C) as a consequence of (i) only land use change, (ii) only climate change and (iii) the combined effect of both land use change and climate change, considering 8 different climate models/scenarios.

Model - Scenario	*(i) only LUC - no CLC*		*(ii) only CLC - no LUC*		*(iii) LUC and CLC*	
	*abs. diff. (Mton)*	*rel. diff. (%)*	*abs. diff. (Mton)*	*rel. diff. (%)*	*abs. diff. (Mton)*	*rel. diff. (%)*
ARPEGE B1	−93.0	−2.35	−698.8	−17.69	−774.4	−19.60
ARPEGE A1B	−93.0	−2.35	−992.1	−25.11	−1060.2	−26.83
ARPEGE A2	−93.0	−2.35	−1124.9	−28.47	−1190.1	−30.12
CNCM33_1 A1B	−100.2	−2.48	−759.6	−18.77	−834.9	−20.63
DMIEH5C_2 A1B	−100.5	−2.43	−1152.9	−27.82	−1220.8	−29.46
HADGEM2_1 A1B	−100.2	−2.46	−1013.9	−24.88	−1082.7	−26.57
MPEH5C_1 A1B	−100.4	−2.45	−1089.7	−26.61	−1157.6	−28.26
MPEH5C_2 A1B	−98.7	−2.43	−899.4	−22.10	−970.5	−23.85
*Average*	*−97.4* ± *3.7*	*−2.41* ± *0.05*	*−966.4* ± *167.7*	*−23.93* ± *4.04*	*−1036.4* ± *164.2*	*−25.66* ± *3.94*
