# Supplementary material for: Future C loss in mid-latitude mineral soils: climate change exceeds land use mitigation potential in France
Source: Sci Rep. 2016 Nov 3;6:35798. doi: 10.1038/srep35798 (PMC5093904; doi:10.1038/srep35798)
Supplement: Supplementary Information [file srep35798-s1.pdf]

Future C loss in mid-latitude mineral soils: climate change exceeds land use mitigation potential in France.

Jeroen Meersmans[1*], Dominique Arrouays[2], Anton J.J. Van Rompaey[3], Christian Page[4], Sarah De Baets[1], Timothy A. Quine[1]

[1]Geography Department, College of Life and Environmental Sciences, University of Exeter, Exeter, UK

[2]Infosol Unit, INRA, Orleans, France

[3]Geography and Tourism Research Group, Department Earth and Environmental Sciences, University of Leuven, Leuven, Belgium

[4]Sciences de l'Univers - CERFACS, Toulouse, France.

*Corresponding author: j.meersmans@exeter.ac.uk

Supplementary Information: Land Use Change Modelling (France 1990 - 2100)

Spatial analysis of independent variables LU logistic regression

The spatial distribution (by region) of the variables selected for the logistic regression, predicting the probability of the occurrence of a certain land use, with annotation of the level of significance of the associate parameters, are given in figures 1-4, i.e. for the topographical (Fig. 1), climate (Fig. 2), soil type (Fig. 3) and socio-economic (Fig. 4) variables, respectively.

(i) Topography (Fig. 1)

The likeliness of the occurrence of cropland is generally highest on flat or gently sloping terrains. Moreover, in the South and East of France cropland tends to occur most frequently at lower altitudes and more in particular in the South-East of France on south orientated hillslopes. Equally, the probability to find grasslands is highest on flat or gently sloping terrains in mountainous areas in the East as well as on south oriented slopes in the South-East. Whereas the likeliness for the occurrence of forest is all over France highest on steep sloping terrains and more precisely on north orientated slopes in the South and East of France. In addition, in the North of France forest tends to occur mostly at higher altitudes while in the South of France this is at lower altitudes. The occurrence of vineyards and orchards in the North of France is highest on steeper slopes, while in the South this is highest at non-steep slopes at lower altitudes, with in general all over France a slight preference for south orientated slopes. Urban development has been found to occur most often at lower altitudes in the North and East of France and on non-steep sloping areas in the South-West, Centre and East of France (Fig. 1).

(ii) Climate (Fig. 2)

Croplands are most likely to be found in relatively dry areas in the Northern and Central parts of France and in relatively cool areas in the South, Eastern and Central parts of the country, whereas in the West and South-West of France croplands tend to occur more frequently under relatively humid and/or warm climatological conditions. Grasslands have in general the highest likeliness to occur in relatively humid and cool areas. Forest seems most likely to be found in relatively humid and warm areas, except for the West of France, where they are found in dry and cold areas. Vineyards and orchards generally occur in relatively dry areas (except in the most South-Eastern part of the country). In addition in the central part of

France vineyards and orchards are more frequently found in relatively warm areas. The relation between the occurrence of urban land use and climate variables is unclear, although in the South-West, urban areas tend to be found more often in relatively warm areas (Fig. 2).

(iii) Soil Type (Fig. 3)

Overall, the likeliness of occurrence of cropland tends to be highest on silty soils. In the West (except Brittany) croplands are less likely to be found on clay rich soils characterized by high bulk densities (e.g. alluvial plains of large rivers reaching coastal areas). In addition, in the more mountainous regions in France in the East and South-East (+ Brittany), croplands are more likely to be found on soils characterized by low stone contents, while in most other regions (not mountainous) this is the opposite (i.e. higher probability on soils with rather high stone content). The link between soil type and the spatial pattern of the probability of occurrence of grassland and forest is less clear. Nevertheless, the results indicate that grassland seems most often located on very fine textured soils (i.e. high clay content or low geometric mean particle size (Dg)) as well as on soils characterized by low stone content (especially in the North and Central part of France), whereas forests are more likely to be found on very fine textured soils (i.e. low Dg and high clay content) in the South-East of France and on more medium-fine - coarse textured soils in other regions (i.e. higher Dg and high silt content). In addition, forests appear more frequently on soils characterized by high stone contents in mountainous regions in the South-East of France as well as in the West, whereas in most other regions forests are more often located on soils characterized by low stone contents. Finally, vineyard and orchards are more likely to be found on soils characterized by high stone contents (South-East) and/or low clay contents (South of France). The relationship between soil type and urban land use is unclear, although in general urban

areas are found on soils characterized by high Dg (i.e. sandy soils), the latter can be observed in particular in the West of France (Fig. 3).

(iv) Socio economic (Fig. 4)

Everywhere in France, unsurprisingly, urban areas have a high probability to be located in areas characterized by a high population potential, but nevertheless no clear overall trend could be found with employment potential. In addition, a clear West-East distinction can be observed as regards the influence of the distance to a main route on the probability of occurrence of urban areas, i.e. in the East of France urban areas are most likely to be found close to routes, whereas in the West of France the distance to the main route has not been selected as explanatory variable in the logistic regression. Furthermore, the likeliness to find cropland is highest in areas characterized by low population density in most of the regions situated in the Northern and Central parts of France and in areas characterized by low employment potential in the South of France. Grasslands are most likely to be found in areas characterized by low employment potential in most regions situated in the Central and Eastern parts of France and in areas characterized by high population density in the East and South of France. The results indicate that forest in the East of France are more likely to occur further away from main routes while in the most Western part of the country closer to main roads. Furthermore, in the South-West forest are more likely to be found in areas characterized by low population potential but rather high employability potential. Relationships between the considered socio-economic variables and the likeliness of occurrence of vineyard and orchards are less clear, although in the North, vineyard and orchards tend to be found closer to the main route and in the South further away from the main route (Fig. 4).

Validation

The resulting land use change predictions were validated by comparing (on a pixel by pixel basis) the predicted land use map for 2000 with the land use observations as represented in the CORINE land cover map of 2000. The overall accuracy, i.e. relative proportion of pixels that has been predicted correctly, varies depending on the region between 0.975 and 0.997 (with an average value and standard deviation of 0.988 ± 0.006). Average land use type specific accuracy values are 0.987 ± 0.008, 0.968 ± 0.063, 0.996 ± 0.003 and 0.974 ± 0.035 and 0.947 ± 0.018 for cropland, grassland, vineyard & orchard and urban, respectively. After taking the chance of agreement into account, the kappa index of agreement was calculated for each region and varies between 0.959 and 0.996 (with an average value and standard deviation of 0.980 ± 0.011). Figure 5 gives the spatial distribution of these validation measures. This figure shows that cropland and forest are characterized by very high accuracies all over the French territory, whereas grassland and vineyard & orchards have somewhat lower accuracy values in the Central and North-Western part of France and urban in the Southern and Western part of France. Both of these trends are reflected in the maps (Fig. 5) showing the overall accuracy and Kappa index of agreement values.

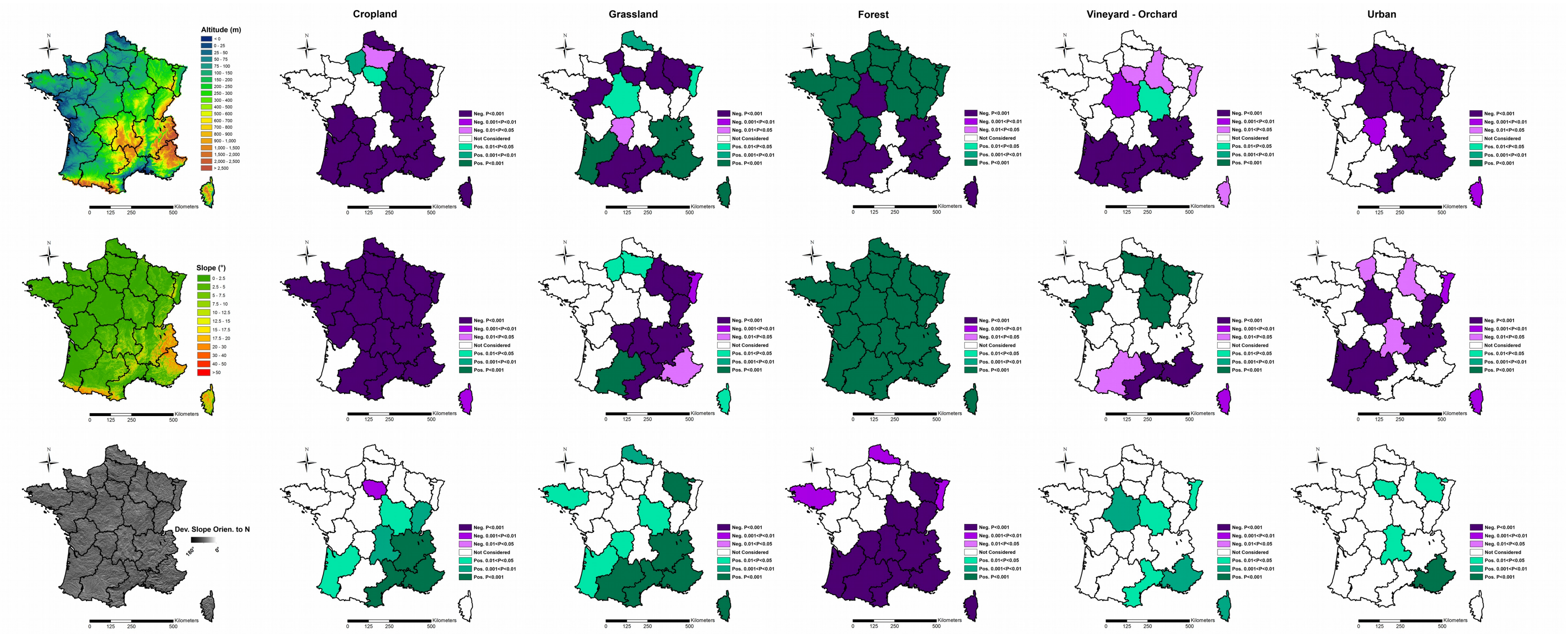

Figure 1: Spatial distribution (by region) of the topographical variables used for the logistic regression, i.e. predicting the probability of the occurrence of the 5 considered land uses, with annotation of the level of significance of the associate parameters. The maps were generated using ArcGIS 10.1 (ESRI, Redlands, CA, USA: http://www.esri.com/software/arcgis).

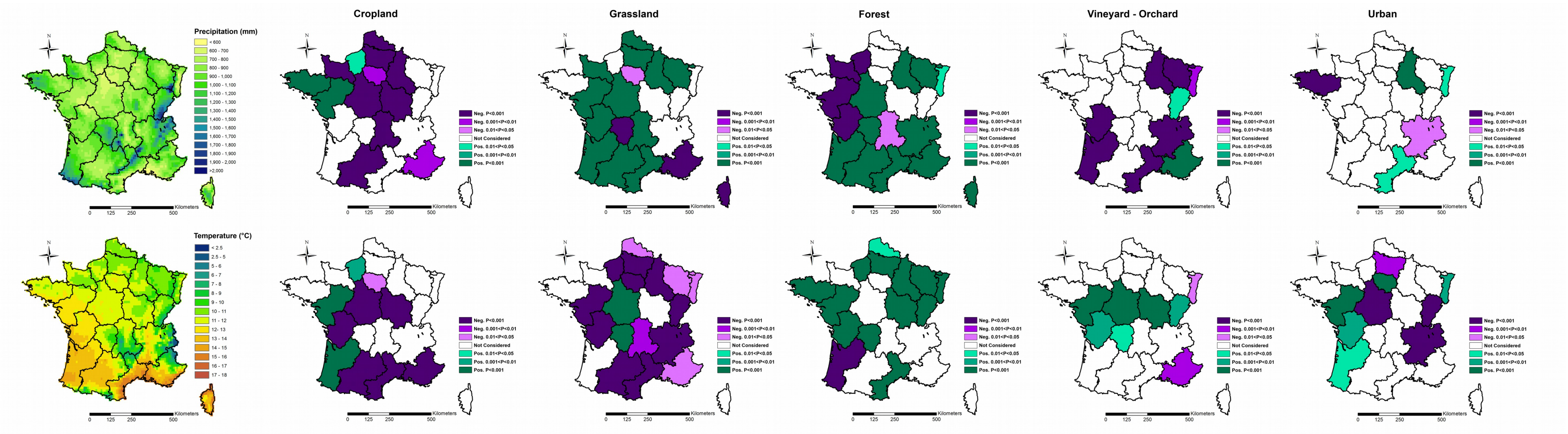

Figure 2: Spatial distribution (by region) of the climate variables used for the logistic regression, i.e. predicting the probability of the occurrence of the 5 considered land uses, with annotation of the level of significance of the associate parameters. The maps were generated using ArcGIS 10.1 (ESRI, Redlands, CA, USA: http://www.esri.com/software/arcgis).

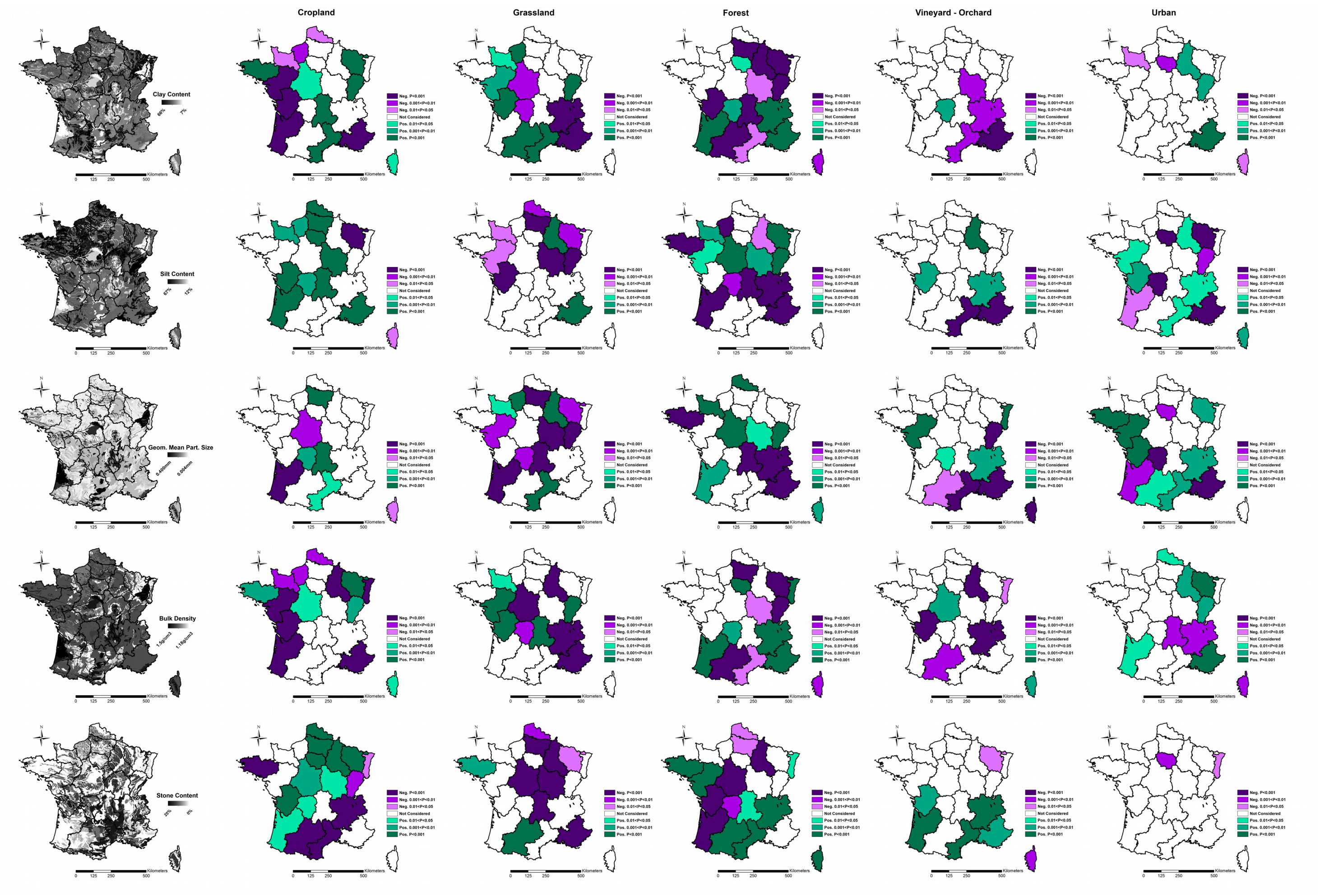

Figure 3: Spatial distribution (by region) of the soil type variables used for the logistic regression, i.e. predicting the probability of the occurrence of the 5 considered land uses, with annotation of the level of significance of the associate parameters. The maps were generated using ArcGIS 10.1 (ESRI, Redlands, CA, USA: http://www.esri.com/software/arcgis).

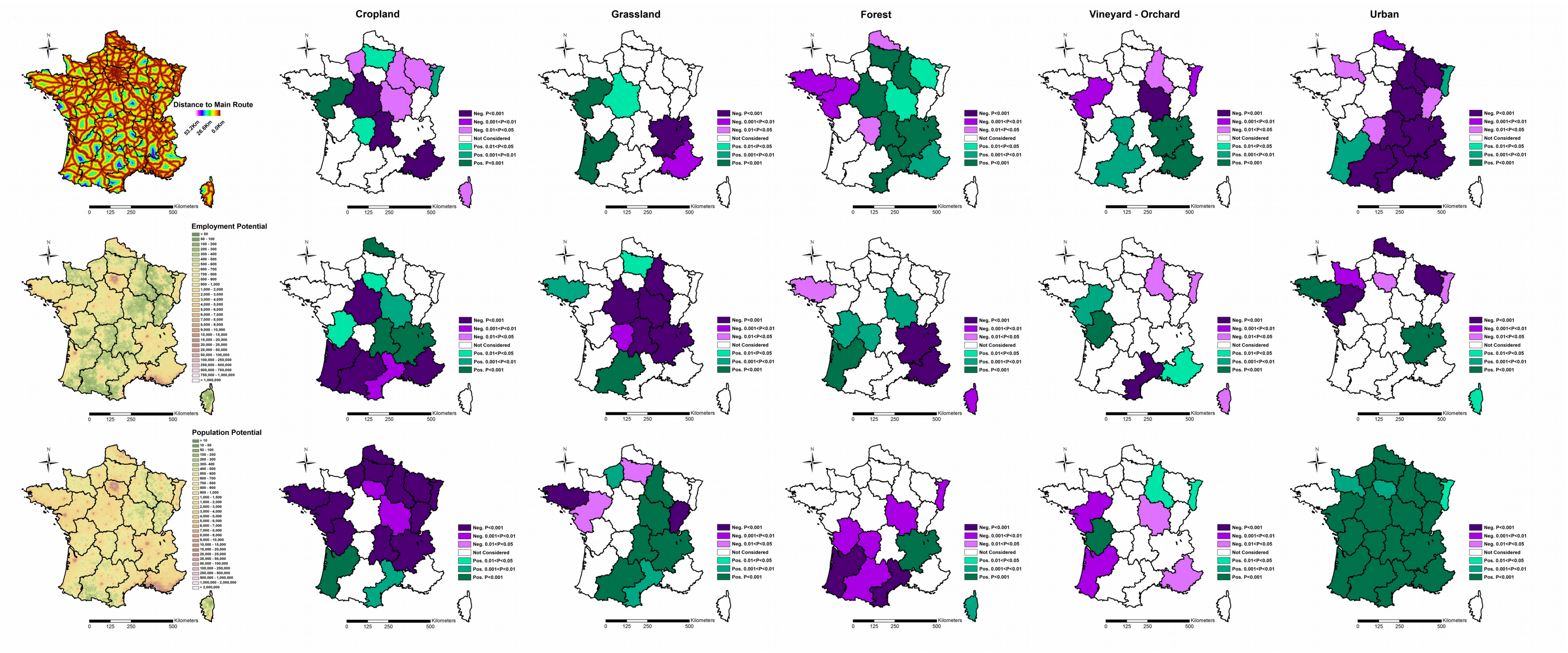

Figure 4: Spatial distribution (by region) of the socio-economic variables used for the logistic regression, i.e. predicting the probability of the occurrence of the 5 considered land uses, with annotation of the level of significance of the associate parameters. The maps were generated using ArcGIS 10.1 (ESRI, Redlands, CA, USA: http://www.esri.com/software/arcgis).

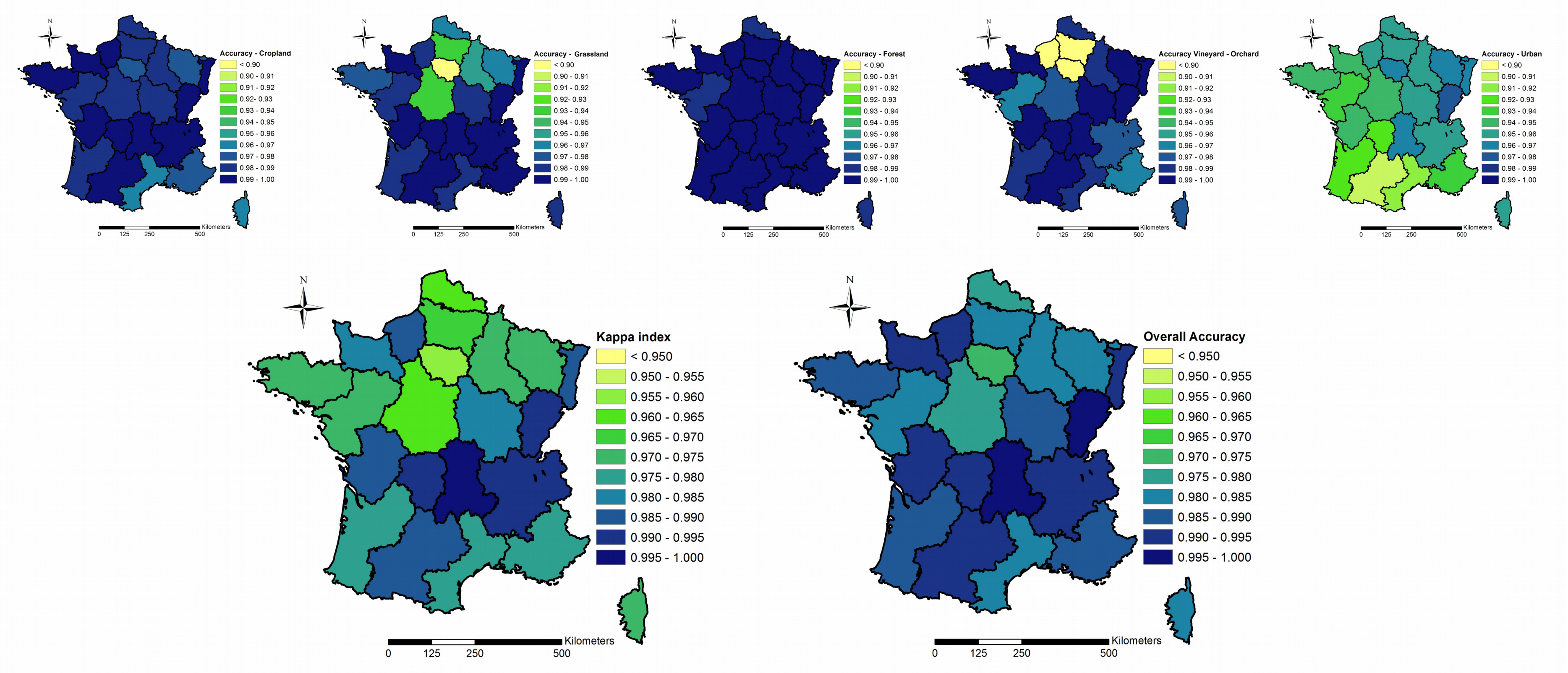

Figure 5: Spatial distribution (by region) of the considered validation measures of the logistic regression used to predict the probability of the occurrence of the 5 considered land uses, i.e. overall and land use type specific accuracy values & Kappa index of agreement. The maps were generated using ArcGIS 10.1 (ESRI, Redlands, CA, USA: http://www.esri.com/software/arcgis).
